# A novel screw-and-cement pile technique for Harrington II/III periacetabular lesions: technical note and short-term outcomes

**DOI:** 10.3389/fonc.2026.1696359

**Published:** 2026-02-24

**Authors:** Leming Mou, Hongfei Li, Jiawei Xu, Changgang Zhu, Siying Li, Jingyu Zhang, Yancheng Liu, Yongcheng Hu, Dengxing Lun

**Affiliations:** 1 WeiFang People’s Hospital, Shandong Second Medical University, Weifang, Shandong, China; 2 Department of Anesthesiology, Umass Chan Medical School, Springfield, MA, United States; 3 Shandong Weigao Orthopedic Device Co., Ltd., Weihai, Shandong, China; 4 Bone Tumor and Soft Tissue Oncology, Tianjin Hospital, Tianjin University, Tianjin, China

**Keywords:** acetabular reconstruction, Harrington technique, periacetabular metastases, screw-and-cement pile technique, total hip arthroplasty

## Abstract

**Background:**

Periacetabular metastases, particularly Harrington type II/III lesions, frequently lead to acetabular insufficiency, femoral head migration, and severe pain. We developed a simplified screw-and-cement pile technique using readily available screws, left partially proud, to interlock with polymethylmethacrylate (PMMA), supporting a standard polyethylene cup through a single conventional approach to reconstruct the damaged acetabular structure.

**Methods:**

We performed a retrospective, two-center case series involving six consecutive patients, three with Harrington type II lesions and three with Harrington type III lesions. Surgical procedures included curettage, screw placement toward the acetabular dome, with additional screws to the pubic and ischial rami as needed, PMMA embedding, and implantation of a standard polyethylene cup via a posterolateral approach. Outcomes assessed included surgical invasiveness, pain (VAS), limb function, complications, and oncologic outcomes.

**Results:**

The mean age of the patients was 65.8 ± 6.1 years, with a mean follow-up of 13.3 ± 7.9 months. The mean operative time was 148.3 ± 27.9 minutes, and average blood loss was 650.0 ± 367.4 mL. VAS scores improved from 7.3 ± 0.5 preoperatively to 1.0 ± 0.6 at 1 month (p < 0.001). The Harris Hip Score (HHS) increased from 30.7 ± 5.6 preoperatively to 81.7 ± 1.9 at 1 month and 86.2 ± 2.7 at final follow-up (both p < 0.001). The MSTS-93 score averaged 20.7 ± 1.0 at 1 month and 22.3 ± 0.8 at final follow-up. No postoperative complications occurred. All patients were alive at the last follow-up, with no evidence of local progression, and all received disease-specific systemic therapy.

**Conclusion:**

In short-term follow-up, the screw-and-cement pile technique for Harrington II/III acetabular defects provided rapid pain relief and functional improvement without early complications. Featuring a simple approach and low-cost implants, it is a viable palliative option for achieving immediate stability and symptom control. Longer-term studies are warranted to evaluate its durability.

## Introduction

Osseous metastasis affects up to 70% of patients with advanced cancer (1), with the pelvis being the second most common site of bony metastasis, following the spine (2). Although radiation therapy, chemotherapy, and bisphosphonate medications have played significant roles in the treatment of bone metastases (3), pelvic metastases, particularly those near the acetabulum, are frequently associated with pathological fractures and migration of the femoral head. This leads to severe pain and reduced mobility, making surgical reconstruction the cornerstone of treatment to restore joint function and alleviate symptoms, thereby improving the quality of life for affected patients (4, 5). However, reconstructing the acetabulum remains a significant challenge for orthopedic surgeons due to its complex anatomy, high biomechanical demands, and the necessity to restore both load-bearing capacity and joint mobility (6). The proximity of critical neurovascular structures and the requirement for durable fixation further compound the difficulty, particularly in patients with compromised bone integrity. Over the decades, various reconstructive strategies, including saddle prostheses, custom-made pelvic replacements, total hip arthroplasty (THA) with reinforcement cages, massive allografts, and resection arthroplasty, have yielded mixed results, with failure rates ranging from 0% to 29% (2, 4, 7–15), reflecting the inherent difficulty of these procedures and variability in patient profiles.

Harrington introduced a classification system for metastatic periacetabular lesions and proposed corresponding reconstruction strategies (16). Specifically, for Harrington type III lesions, which are characterized by destruction of the medial wall and acetabular dome, his technique involved placing multiple Steinmann pins from the ilium to support a cemented acetabular cup. Numerous modifications have since evolved, aiming to improve construct stability and reduce complications (8, 17, 18). However, these techniques often require extensive exposure beyond the traditional hip arthroplasty approach, involving either dual surgical windows or extended incisions. This contributes to prolonged operative time, increased blood loss, and a steeper learning curve, limiting their adoption, especially in centers lacking advanced tumor reconstruction expertise. Furthermore, some versions rely on flanged or constrained cups and customized cages, which are not always readily accessible and may impose financial burdens (2).

A simplified screw-and-cement reinforcement technique has been introduced, aiming to provide mechanical stability using readily available materials while minimizing surgical invasiveness. This method avoids the use of Steinmann pins and instead employs multiple screws anchored into the ilium, reinforced with polymethylmethacrylate (PMMA), to support a standard polyethylene cup. This intra-articular construct has demonstrated promising outcomes in selected reports, particularly for Harrington type II and III lesions, offering a potentially less invasive and more economical alternative (19, 20). Despite these advantages, detailed biomechanical rationale, screw placement strategies, and surgical technique descriptions remain scarce in current literature. Most studies reporting on this technique do not provide a standardized protocol or step-by-step guidance, which hinders widespread clinical adoption.

According to previous studies, the primary load-bearing regions of the pelvis include the acetabular dome and the incisura ischiadica area, with the pubic bone contributing to a lesser extent (21, 22). Based on this established biomechanical understanding, we refined this method and named it the screw-and-cement pile technique to highlight its underlying principle, which is akin to pile driving in foundation engineering, where screws act as piles anchored in cement to restore structural integrity. In this study, we retrospectively analyzed cases from two bone tumor centers using this technique with a standard polyethylene cup to treat Harrington Type II and III lesions. We aimed to define the screw trajectories and surgical workflow of this technique while evaluating its clinical outcomes, proposing it as a practical and reproducible solution for periacetabular defects where complex implants or extensive approaches are not feasible.

## Patients and methods

### Inclusion and exclusion criteria

We retrospectively reviewed medical records from two bone tumor centers between October 2023 and May 2025. Inclusion criteria were as follows: (i) histologically confirmed metastatic lesions involving the acetabulum, classified as Harrington type II or III; (ii) reconstruction using the screw-and-cement pile technique combined with a standard polyethylene cup; (iii) expected survival time of more than six months. Exclusion criteria were: (i) patients with primary pelvic tumors or solitary metastases requiring en bloc resection and custom prosthetic reconstruction; (ii) patients who were unable to complete follow-up or had incomplete medical records.

All procedures were performed by experienced orthopedic oncologists specializing in periacetabular reconstruction. Institutional review board approval was obtained from both participating centers, and informed consent was obtained from all patients prior to surgery.

### Preoperative evaluation and classification

Preoperative imaging comprised anteroposterior pelvis radiographs, thin-slice CT to define cortical loss and intrapelvic extension, and MRI to evaluate marrow and soft-tissue involvement. Lesions were classified according to the Harrington system, with explicit documentation of medial wall and acetabular dome compromise to guide reconstruction. Surgical candidacy was determined by a multidisciplinary team considering overall oncologic status, Karnofsky Performance Status (KPS) (23) and revised Tokuhashi scores (24), and an anticipated survival greater than six months.

### Surgical technique

**Figure 1:**
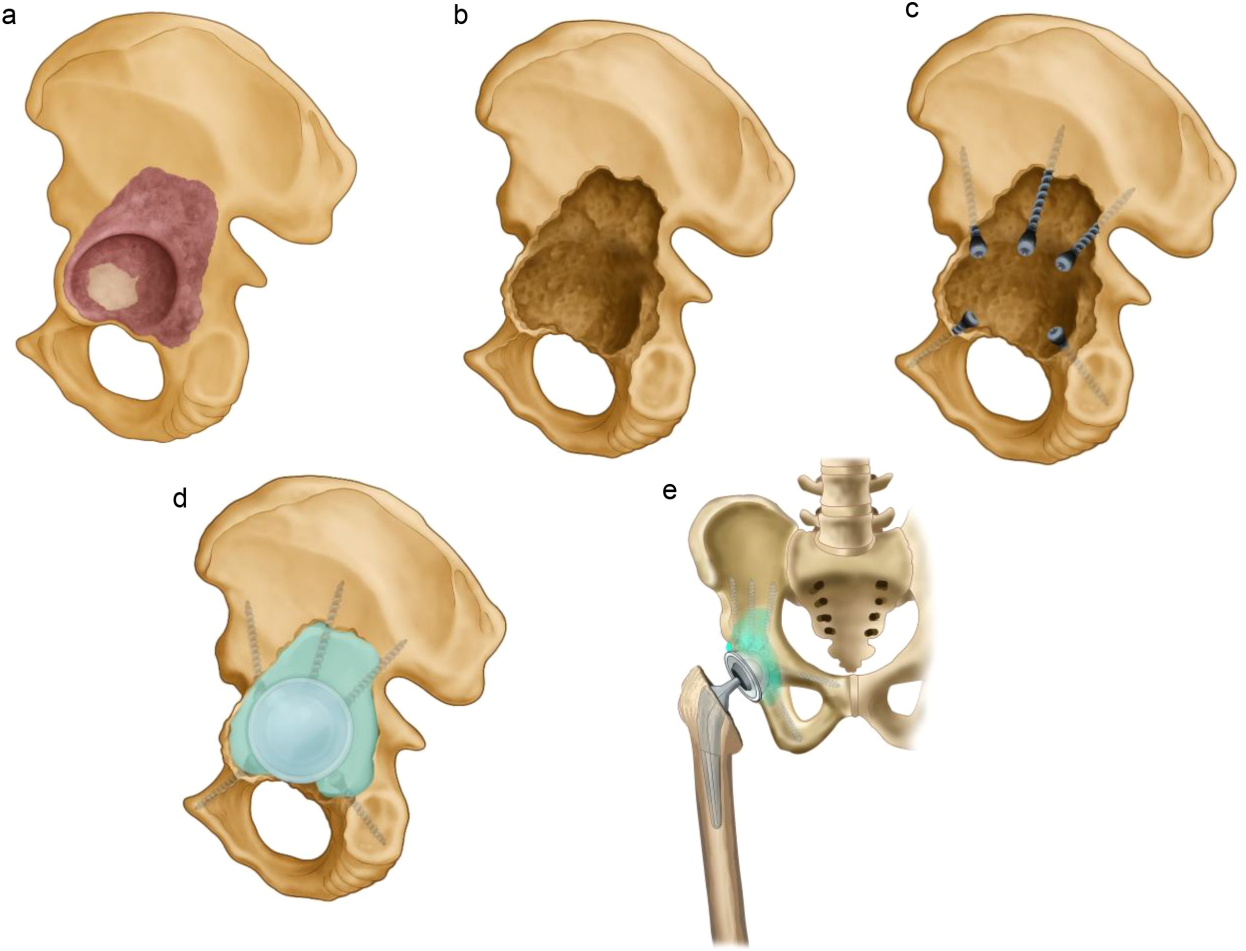
Workflow schematic of the Screw-and-Cement Pile Technique. **(a)** Variable tumor involvement of the acetabular dome, posterior column, and medial wall; **(b)** After intralesional curettage, cavitary/segmental defects enlarge the acetabulum and distort its native contour; **(c)** Three cancellous screws are directed toward the acetabular dome, with priority given to areas of thicker bone at the anterior and posterior iliac regions. The number of screws used is determined based on the clinical situation, and additional screws may be placed into the superior pubic and ischial rami as needed. Screws are intentionally left partially proud to function as “piles,” and a polyethylene trial cup is used to ensure that the screw heads do not interfere with proper cup seating; **(d)** Polymethylmethacrylate (PMMA) is introduced to fill the defect and embed the screws, followed by implantation of a polyethylene cup with appropriate inclination and anteversion; stability is maintained until polymerization; **(e)** The femoral side is prepared in standard fashion, an appropriate femoral stem and head are implanted, and the hip is reduced.

All procedures were performed under general anesthesia, with the patient placed in a lateral decubitus position. A standard posterolateral approach was used in all cases, providing sufficient exposure to the acetabulum and proximal femur without the necessity for extended or dual incisions. A schematic of the surgical workflow is shown in **Figure 1**.

Following femoral head dislocation and osteotomy, the acetabulum was exposed and the residual articular cartilage was removed. Intralesional curettage was performed to excise tumor tissue and any tumor-involved bone that had lost structural integrity. When tumor-involved bone retained sufficient mechanical strength, it was preserved to maintain support; in these regions, microwave ablation was applied for local tumor devitalization. The cavity was then systematically inspected to assess the integrity of the medial wall and the acetabular dome in order to guide reconstruction. Two or three cancellous bone screws (diameter: 4.5–6.5 mm) were inserted toward the acetabular dome, preferentially anchored in regions of structurally sound and thicker bone. In cases where three screws were used, the first was directed toward the incisura ischiadica, a key site for axial load transmission within the pelvis. The second screw was targeted at the central portion of the acetabular dome, the primary weight-bearing region during standing and gait. The third screw was oriented toward the anterior inferior iliac spine. Additional screws were placed into the pubic and ischial rami when necessary. Intraoperative fluoroscopy, including anteroposterior, lateral, inlet, outlet, iliac oblique and obturator oblique projections, was employed to guide screw placement and prevent inadvertent bone penetration. All screws were inserted from within the acetabulum toward the surrounding bone. Importantly, the screws were intentionally left protruding from the bone surface (e.g., 3-5 mm or more, depending on the defect) to function as “piles,” securing the bone cement and forming a stable, anchored scaffold capable of resisting multidirectional mechanical stresses.

Polymethylmethacrylate (PMMA) bone cement was then introduced to fill the defect, encapsulating the screw heads and occupying residual voids to reconstruct a stable neo-acetabulum. A standard polyethylene cemented acetabular cup was pressurized into the cement bed with a target orientation of 40–45° of inclination and 15–20° of anteversion and was held in position under steady manual pressure until the cement had fully polymerized. Femoral reconstruction was performed using standard total hip arthroplasty techniques. In patients with proximal femoral involvement, a limited proximal femoral resection was carried out, followed by implantation of a femoral component incorporating a proximal reconstruction segment. After reduction, hip stability was evaluated through a full arc of motion. Closed-suction drains were placed in all cases, and the incision was closed in a layered fashion.

### Postoperative protocol

All patients received antibiotic prophylaxis and thromboprophylaxis postoperatively. Partial weight-bearing was allowed from postoperative day 2–3, progressing to full weight-bearing over 1 week. Adjuvant radiotherapy and systemic treatment were administered as per oncologic protocols.

### Outcome assessment

#### Surgical invasiveness

Operative time (minutes) and intraoperative blood loss (ml) were recorded for each procedure to evaluate surgical invasiveness.

#### Pain

Pain severity was assessed using a 10-point Visual Analog Scale (VAS; 0 = no pain, 10 = worst imaginable pain) preoperatively, and at the 1-month follow-up.

#### Functional outcome

Limb function was evaluated with the Musculoskeletal Tumor Society score (MSTS-93), which was collected at 1 month postoperatively, and at final follow-up. Hip function was assessed using the Harris Hip Score (HHS; 0–100) preoperatively, at 1 month postoperatively, and at final follow-up.

#### Postoperative complications

Complications were categorized according to the Henderson classification (25) for limb-salvage reconstruction failures: type 1—soft-tissue failure; type 2—aseptic loosening; type 3—structural failure; type 4—infection; type 5—tumor progression.

#### Oncologic outcomes

Postoperative oncologic management and surveillance were coordinated with the multidisciplinary oncology team. Oncologic status was documented at scheduled postoperative outpatient follow-up visits.

### Statistical analysis

Analyses were performed using R (version 4.4.2; R Foundation for Statistical Computing, Vienna, Austria). Descriptive statistics (frequency, percentage, mean, standard deviation) were calculated. Continuous variables were compared using Student’s t - test for independent samples or paired t-tests for within-patient comparisons, as appropriate. Statistical significance was set at two-sided p < 0.05.

## Results

### General results

Six patients (2 men, 4 women) with a mean age of 65.8 ± 6.1 years (range, 59–75) underwent reconstruction. Primary diagnoses were lung cancer (n = 2), renal cell carcinoma (n = 1), breast cancer (n = 1), esophageal cancer (n = 1), and Gorham–Stout disease (n = 1). Preoperative performance status was assessed with a KPS score of 75 ± 5.5 (median 75; range, 70–80), and the baseline Tokuhashi score was 10.7 ± 2.0 (median 10; range, 9–13). According to the Harrington classification, three patients were type II and three were type III. The mean clinical follow-up was 13.3 ± 7.9 months. Detailed results are provided in **Table 1**.

**Table 1:** presents data of 6 patients.

NO.	Age	Sex	Diagnosis	KPS score	Tokuhashi score	Harrington classification	Operation time (min)	Intraoperative blood loss (ml)	Follow-up (months)	Complications	VAS score (Preoperative)	VAS score (1-month postoperative)	MSTS score (1-month postoperative)	MSTS score (last follow-up)	Harris score (Preoperative)	Harris score (1-month postoperative)	Harris score (last follow-up)
1	59	M	Renal cell carcinoma	70	11	III	150	800	22	None	8	1	20	22	23	81	85
2	63	W	Gorham-Stout disease	70	–	III	200	1200	19	None	8	1	19	21	25	80	85
3	75	W	Breast cancer	80	13	III	120	200	16	None	7	0	22	23	31	85	91
4	66	W	Lung cancer	80	9	II	130	800	16	None	7	1	21	23	35	82	87
5	61	M	Esophageal cancer	80	9	II	150	300	4	None	7	2	21	22	37	80	83
6	71	W	Lung cancer	70	9	II	140	600	3	None	7	1	21	23	33	82	86

M, men; W, women; KPS, Karnofsky Performance Status; VAS, Visual Analog Scale; MSTS, Musculoskeletal Tumor Society.

### Surgical invasiveness

The mean operative time was 148.3 ± 27.9 minutes. The mean intraoperative blood loss was 650.0 ± 367.4 mL.

#### Pain

VAS pain scores improved from 7.3 ± 0.5 preoperatively to 1.0 ± 0.6 at 1 month postoperatively (p < 0.001). Improvement was observed in both type II and type III cohorts.

#### Functional outcomes

The mean MSTS-93 score was 20.7 ± 1.0 at 1 month postoperatively and 22.3 ± 0.8 at final follow-up. HHS improved from 30.7 ± 5.6 preoperatively to 81.7 ± 1.9 at 1 month postoperatively (p < 0.001) and to 86.2 ± 2.7 at final follow-up (p < 0.001). All patients achieved a good–excellent HHS (≥80) at final follow-up.

#### Postoperative complications

**Figure 2:**
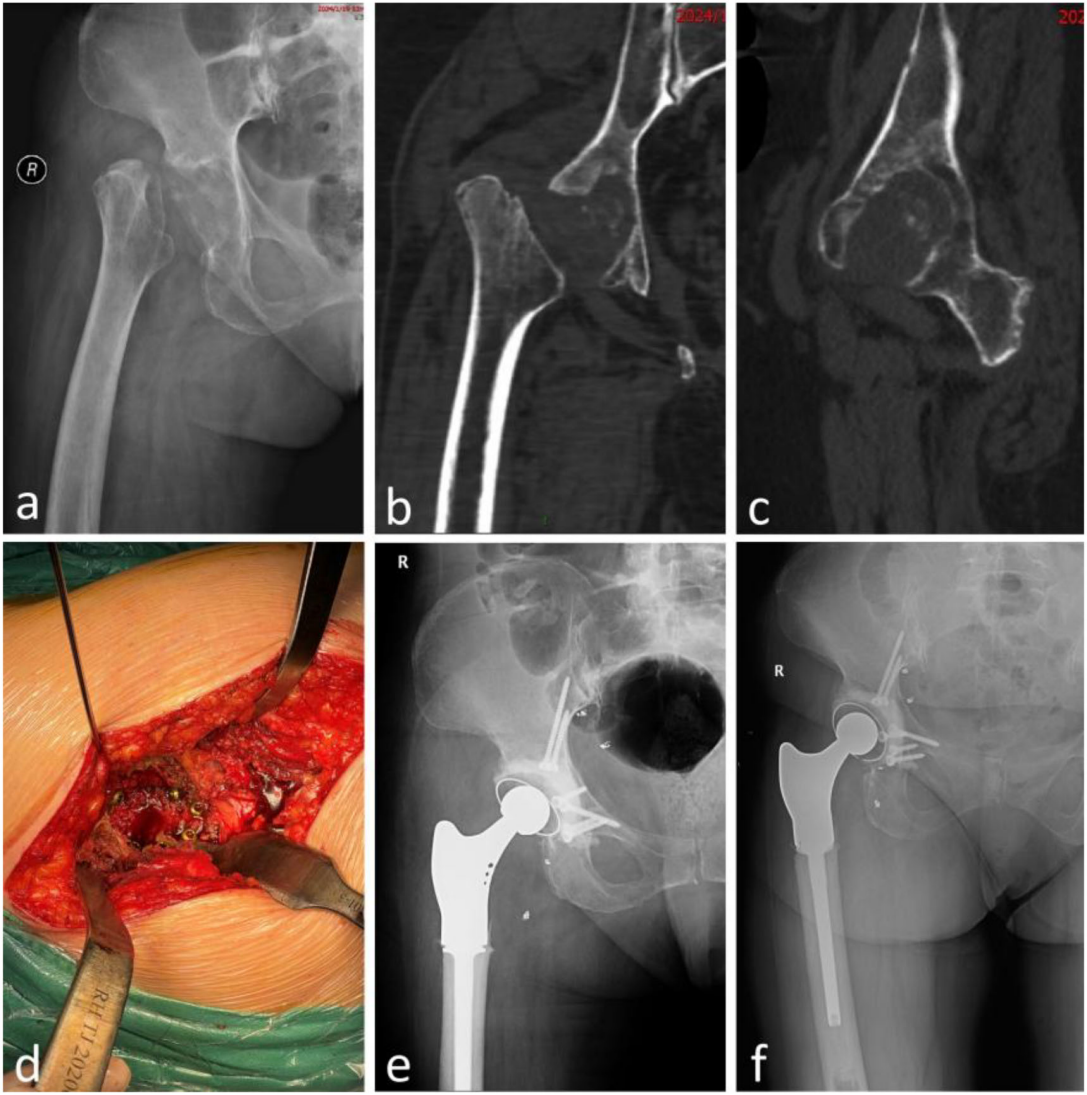
Patient 2 with Gorham–Stout disease underwent acetabular reconstruction using the screw-and-cement pile technique with a standard polyethylene cemented cup, together with total hip arthroplasty and partial proximal femoral replacement. **(a)** Preoperative anteroposterior (AP) hip joint radiograph demonstrating extensive acetabular destruction with loss of normal contour and absence of the femoral head and neck; **(b, c)** preoperative computed tomography confirming the same defects; **(d)** intraoperative view showing multiple screws inserted from within the acetabulum and directed radially outward, intentionally left partially proud to function as “piles”; **(e)** postoperative AP hip joint radiograph; **(f)** AP hip joint radiograph at 16 months demonstrating maintained component position without screw back-out, cup migration, or loosening.

**Figure 3:**
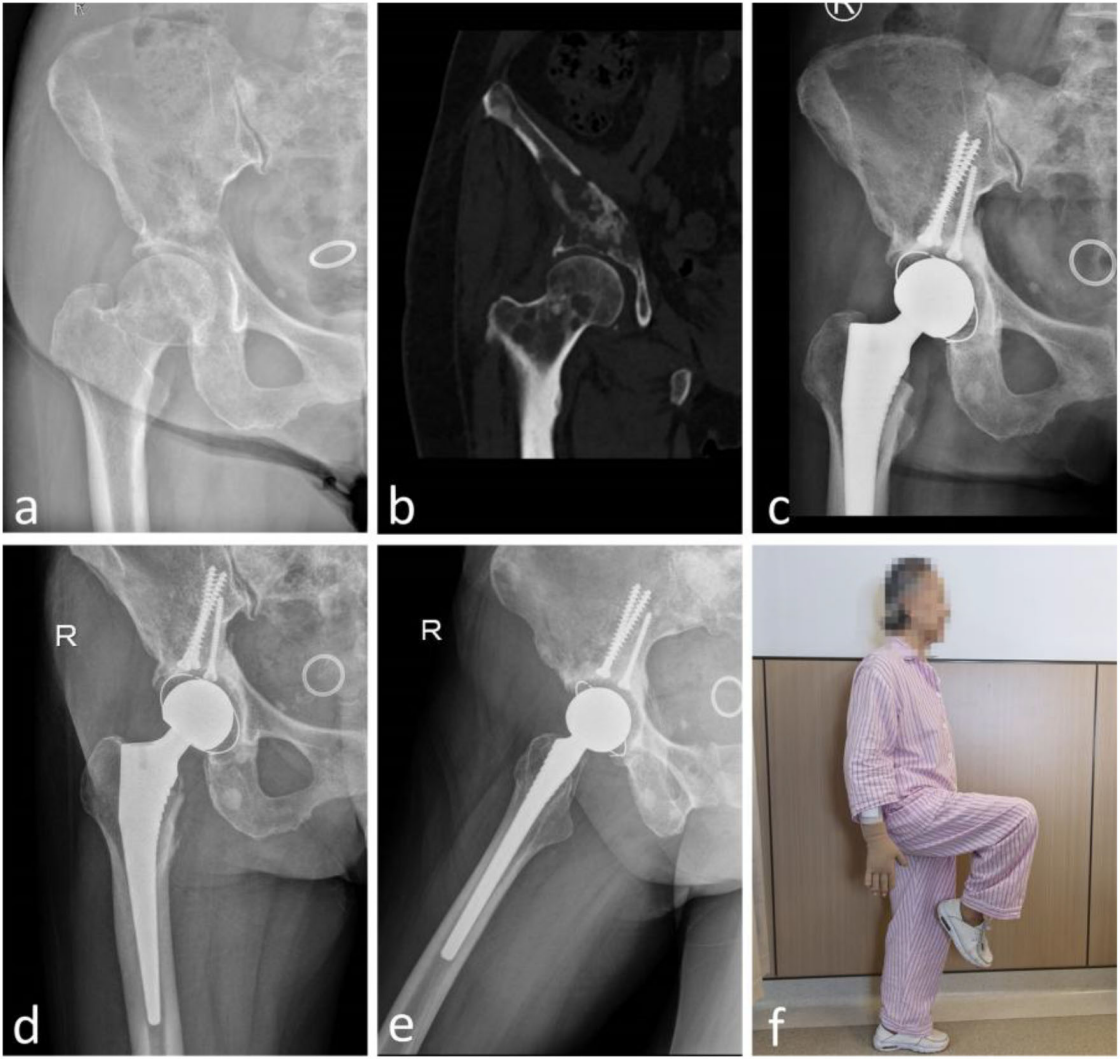
Patient 3 with breast cancer metastasis to the acetabulum treated with Screw-and-Cement Pile reconstruction using a standard polyethylene cemented cup and total hip arthroplasty. Intraoperative microwave ablation was applied to the tumor-involved region preserved at the acetabular dome. **(a)** Preoperative anteroposterior (AP) pelvic radiograph and **(b)** preoperative computed tomography (CT) showing marked lytic destruction of the acetabular roof with involvement of the femoral head and neck; **(c)** AP hip radiograph at 5 months demonstrating well-positioned components without screw back-out, cup migration, or loosening; **(d)** AP and **(e)** lateral hip radiographs at 16 months showing maintained component position without loosening or migration; **(f)** Standing lateral view at 16 months demonstrating good hip flexion.

No postoperative complications were observed through the last follow-up. In one patient (patient 4), radiographs at 11 months demonstrated radiolucent lines at the cement–bone interface without symptoms. No cup migration (>2 mm) or change in inclination (>5°) was detected in any case. Screw positions remained unchanged, and no mechanical loosening was identified. No reoperations were required. Patients 2 and 3 serve as illustrative cases (**Figures 2**, **3**).

#### Oncologic outcomes

All patients received disease-specific systemic therapy after surgery as indicated by the oncology team. For patients requiring chemotherapy, surgery is typically scheduled after the bone marrow suppression induced by chemotherapy has substantially subsided. Postoperatively, the first cycle of chemotherapy is generally initiated following complete wound healing. At last follow-up, all patients were alive. No clinical or radiographic evidence of local progression or recurrence of the acetabular lesion was observed.

## Discussion

The management of periacetabular bone defects secondary to metastatic disease remains a formidable challenge for orthopedic oncologists. Harrington type II and III lesions are often characterized by acetabular structural insufficiency, femoral head migration, and severe pain, prompting surgical reconstruction to restore weight-bearing function and improve quality of life. In this study, we evaluated a simplified screw-and-cement piling technique as a biomechanically rational and resource-efficient alternative to more complex reconstructive methods.

In this series of six patients, we observed a rapid analgesic response within one month following treatment. At the last follow-up, functional recovery was durable, with a mean MSTS score of 22.3. Furthermore, all patients achieved good to excellent hip scores, with HHS ≥80, and no postoperative complications were reported. These outcomes are consistent with the broader literature showing that open reconstruction which re-establishes load transfer across the acetabular dome reliably improves pain and function in appropriately selected patients with metastatic periacetabular disease (26). Our early experience supports the central premise of Harrington-style reconstructions, which is that a mechanically competent bed can be recreated to transmit forces to the remaining structural bone, thereby enabling immediate rehabilitation (16).

Since Harrington’s seminal description of pins-and-cement constructs to bypass dome and medial wall insufficiency, multiple evolutions have been reported (19, 27). In a series of 63 hips, Ghert et al. (7) reported that Harrington-type reconstructions improved mean ECOG performance status scores from 2.6 to 1.1, despite a 17% complication rate including local progression. Marco et al. (8) observed that flanged cup reconstructions were associated with an 11% mechanical failure rate and frequent local tumor progression. Acetabular cage reconstructions carried a 17% dislocation rate in Clayer et al.’s series of 29 patients (10). Percutaneous cementoplasty, evaluated by Maccauro et al. (28) in 30 procedures, provided a mean pain relief duration of 7.3 months in 59% of patients. The systematic review by Brown et al. (29)(2018) of 1700 periacetabular reconstructions reported a 50% overall complication rate across established techniques: Harrington reconstructions (n=415) achieved 67% MSTS score with 8% dislocation; saddle prostheses (n=135) showed high infection (24%) and poor function (57% MSTS); custom and modular implants (n=182 and n=143, respectively) attained 63–69% MSTS scores but incurred >23% infection rates. These conventional reconstruction techniques are notably invasive, typically requiring extended exposures, additional incisions, and substantial soft-tissue dissection. This elevates the risk of infection, increases intraoperative blood loss, and may compromise postoperative functional recovery. Moreover, the frequent reliance on non-standard or custom implants contributes to increased costs and prolonged preoperative waiting periods. Percutaneous cementoplasty provides a minimally invasive and cost-effective option but is primarily suitable for cases with only mild osteolysis and no structural collapse.

In this context, our screw-and-cement piling technique intentionally avoids the use of large rings, cages, and Steinmann pins, instead utilizing readily available cancellous screws left partially proud to serve as “piles.” These interlock with polymethylmethacrylate (PMMA) and support a standard polyethylene cup. All reconstructions were performed through a single conventional posterolateral approach, which simplifies the procedure by incorporating familiar arthroplasty workflows, likely shortening the learning curve and minimizing soft tissue trauma compared to the dual-window or extended exposures often required for pin–cage constructs. This approach significantly reduces surgical invasiveness and lowers the risk of dislocation caused by soft tissue damage. The average blood loss in this study was 650 mL, considerably lower than the 1800 mL and 2790 mL reported in Harrington et al.’s study of 19 patients with Harrington type II and 25 patients with type III acetabular lesions, respectively.

Our early experience supports the central premise of Harrington-style reconstructions, namely that a mechanically competent bed can be recreated to transmit forces to the remaining structural bone and allow immediate rehabilitation (16). Immediate stability is clinically meaningful because many patients prioritize early ambulation and palliation; registry and review data emphasize that timely weight-bearing and symptom control are cardinal goals of surgery in this setting (26). We observed no postoperative complications in this series, and serial imaging showed no cup migration or screw loosening. However, the small sample size and short follow-up limit definitive conclusions regarding safety and durability; larger cohorts with longer observation are needed to validate these findings.

Percutaneous cementoplasty, whether used alone or in combination with ablation, has been reported as a treatment option for periacetabular metastatic disease, providing pain relief for lytic lesions (30). However, systematic reviews caution that while pain control can be achieved, it may not result in durable reconstruction when the weight-bearing dome is structurally compromised (26). Comparative clinical data further suggest that open reconstruction offers greater improvements in both pain and function compared to acetabuloplasty alone in cases of extensive periacetabular disease (31). In both historical and contemporary cohorts, including anatomically based open reconstructions and modified Harrington constructs, clinically meaningful improvements in pain and hip function have consistently been reported among survivors of periacetabular metastases (3, 7). Our series supports these findings, with all patients achieving good to excellent HHS at the last follow-up and a mean MSTS of 22.3, indicating positive functional outcomes.

Balancing surgical trauma with meaningful biomechanical restoration is crucial in palliative care, especially when considering the cost and availability of pelvic oncology implants. In contrast, our Screw-and-Cement Pile technique, characterized as both “effective and inexpensive,” leverages standard tools and implants, making it an attractive option for patients in palliative-intent care. This approach minimizes procedural burden and resource use while maintaining near-term functional outcomes. This technique is indicated for Harrington type II/III acetabular metastases with predominantly osteolytic destruction, where the weight-bearing dome is compromised yet sufficient residual bone stock remains for screw placement, and when the patient’s life expectancy exceeds three months. Conversely, it is contraindicated in cases of primary bone tumors or solitary bone metastases, which require definitive oncologic resection. Furthermore, it offers a single-incision, reproducible solution, aligning with consistent selection principles emphasized in modern reviews, such as multidisciplinary review, expected survival, and patient-centered goals (4). Our data also indicate high patient acceptance, consistent with the philosophy of using accessible, low-cost materials in palliative treatment.

This study is a small, retrospective, two-center series without a control group, with potential selection bias and limited follow-up. Consequently, the data are insufficient to adequately evaluate the long-term durability of the intervention, including critical endpoints such as late implant migration, screw loosening, dislocation, and local recurrence. We did not perform biomechanical testing or prospective head-to-head comparisons with ring/cage or porous metal strategies. Future studies should incorporate biomechanical analyses, enroll larger cohorts, and extend follow-up duration to more rigorously assess durability, complications, and functional outcomes.

## Conclusion

In our early experience, a screw-and-cement piling construct delivered rapid pain relief, good short-term function, and no early complications for Harrington II/III acetabular lesions. Its single conventional approach, short learning curve, and use of readily available, low-cost implants make it well suited to palliative-intent reconstruction when the goals are immediate stability, ambulation, and symptom control. However, longer-term follow-up studies are required to fully evaluate the durability and late outcomes of this technique.

## Data Availability

The original contributions presented in the study are included in the article/supplementary material. Further inquiries can be directed to the corresponding author.
